# Advanced high-resolution chromatographic strategies for efficient isolation of natural products from complex biological matrices: from metabolite profiling to pure chemical entities

**DOI:** 10.1007/s11101-024-09928-w

**Published:** 2024-05-06

**Authors:** Emerson Ferreira Queiroz, Davy Guillarme, Jean-Luc Wolfender

**Affiliations:** 1 School of Pharmaceutical Sciences, University of Geneva, CMU - Rue Michel-Servet 1, 1211 Geneva 4, Switzerland; 2 Institute of Pharmaceutical Sciences of Western Switzerland (ISPSO), University of Geneva, CMU - Rue Michel Servet 1, 1211 Geneva 4, Switzerland

**Keywords:** Natural products, Isolation, Chromatographic calculation, UHPLC-PDA-ESI-HRMS, Semi-preparative HPLC, Gradient transfer, Multi-detection, Metabolomics, Biomarker identification

## Abstract

The isolation of pure compounds from extracts represents a key step common to all investigations of natural product (NP) research. Isolation methods have gone through a remarkable evolution. Current approaches combine powerful metabolite profiling methods for compounds annotation with omics mining results and/or bioassay for bioactive NPs/biomarkers priorisation. Targeted isolation of prioritized NPs is performed using high-resolution chromatographic methods that closely match those used for analytical profiling. Considerable progress has been made by the introduction of innovative stationary phases providing remarkable selectivity for efficient NPs isolation. Today, efficient separation conditions determined at the analytical scale using high- or ultra-high-performance liquid chromatography can be optimized via HPLC modelling software and efficiently transferred to the semi-preparative scale by chromatographic calculation. This ensures similar selectivity at both the analytical and preparative scales and provides a precise separation prediction. High-resolution conditions at the preparative scale can notably be granted using optimized sample preparation and dry load sample introduction. Monitoring by ultraviolet, mass spectrometry, and or universal systems such as evaporative light scattering detectors and nuclear magnetic resonance allows to precisely guide the isolation or trigger the collection of specific NPs with different structural scaffolds. Such approaches can be applied at different scales depending on the amounts of NPs to be isolated. This review will showcase recent research to highlight both the potential and constraints of using these cutting-edge technologies for the isolation of plant and microorganism metabolites. Several strategies involving their application will be examined and critically discussed.

**Graphical abstract:**

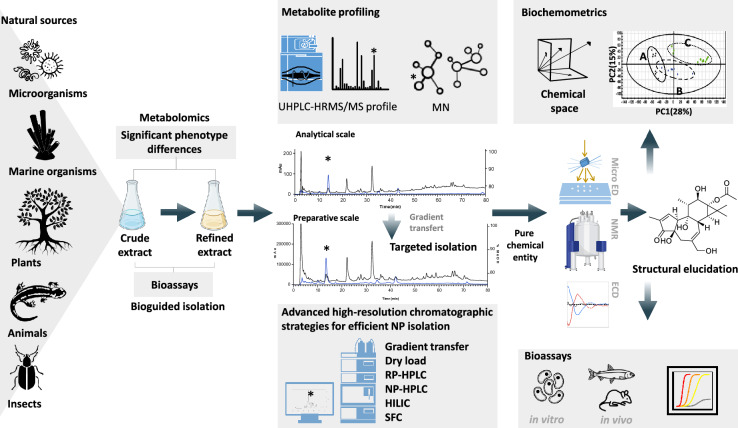

## Introduction

Natural products (NPs) have been shaped in organisms during evolution and represent an unlimited source of inspiration for the discovery of original pharmacophore scaffolds (Newman and Cragg 2020). To produce this interesting chemical diversity, living organisms combine common chemical building blocks in various ways according to specific enzymatic pathways sometimes unique to a genus or species (Nivina et al. 2019). These complex biosynthetic processes result in the production of a diverse range of compounds encompassing a broad spectrum of scaffolds and polarity (Newman and Cragg 2020). Often these compounds occur in a series of closely related structural analogues possessing similar physicochemical properties. Extracts obtained from natural sources (ENs) thus typically contain hundreds of constituents extending over a large range of concentrations. The isolation of pure NPs from ENs of complex composition represents a challenge. In addition, NPs are sometimes present in minute quantities, making it necessary to work with large quantities of raw materials for their isolation.

There are several reasons why pure NPs need to be isolated from their natural biological matrices. Obtaining pure NPs is often a prerequisite for full de novo structural elucidation by nuclear magnetic resonance (NMR), circular dichroism (CD), and X-ray crystallography (X-ray) (Gruene et al. 2018; Kunde and Schmidt 2019). Assessment of the bioactivity of given NPs in bioactivity-guided studies requires high purity to avoid bias interpretation of the pharmacological effects, due to possible residual complexity (Choules et al. 2018). In metabolomics, the unequivocal identification of biomarkers may require the targeted isolation of compounds linked to distinct MS features. This challenge remains due to the recognized difficulties of reliably identifying metabolites in this field.

**Fig. 1:**
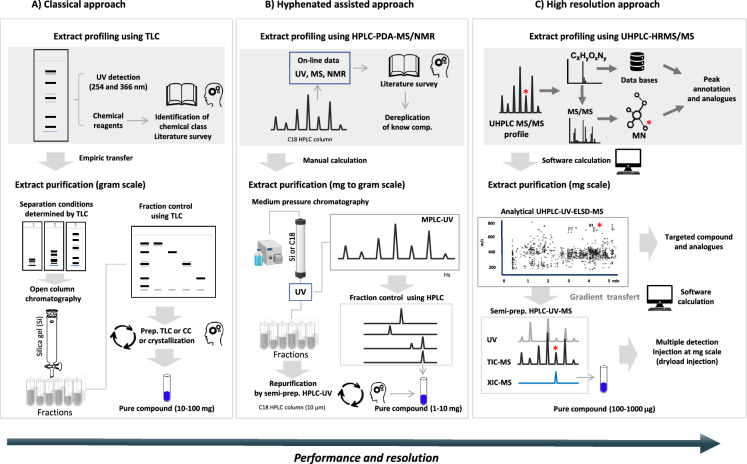
**a** Classical bioguided isolation approach combining TLC, CC, preparative TLC and biological assays. **b** Isolation approach assisted by hyphenated HPLC methods (LC-UV–MS/NMR) and biological assays, isolation using MPLC-UV, and semi-preparative HPLC–UV. **c** Contemporary approach based on advance metabolite profile for the selection of the compound to be isolated, gradient transfer from the analytical to the semi-preparative scale, isolation of the targeted compound by high-resolution chromatography combining dryload injection and multi-detection methods (UV, ELSD and MS) for ideally a single step NP isolation from ENs

Figure 1 gives an overview of the use of preparative chromatography techniques and the various advances that have shaped the isolation of NPs over time.

The conventional approach employed for purifying NPs involves the gram-scale fractionation of extracts using normal phase open column chromatography (CC), typically utilizing silica gel as the stationary phase (Fig. 1a)(Hostettmann et al. 1998). The fractions obtained are then analysed by silica gel thin-layer chromatography (TLC) on the same stationary phase. This process is often combined with bioassay assessment of the extract and the resulting fractions that will drive the isolation process (bioguided isolation)(Alilou et al. 2020a, b; Bucar et al. 2013). As the chromatographic resolution of CC is limited (manually packed columns with large particle size (40–63 µm)), fractions can be further purified by additional preparative CC or TLC steps using a different mobile phase composition to increase selectivity (Alilou et al. 2020a; Hostettmann et al. 1998). Compounds difficult to separate can be purified with high-resolution semi-preparative HPLC. Although efficient, CC fractionation conditions are defined empirically by transferring analytical conditions established by TLC (Fig. 1a). This approach does not ensure good reproducibility of separation, and biological activity throughout the isolation process may be lost due, for example, to irreversible adsorption of NPs onto the silica gel phase.

The use of instruments allowing to work at higher pressure than ambient with stationary phase columns containing smaller particles, enables higher resolution in separations to be achieved in a shorter time (Fig. 1b). In this context, flash chromatography (FC) and medium pressure liquid chromatography (MPLC) have represented interesting alternatives to CC for the separation of gram amounts of extracts with enhanced resolution and, on the other hand, semi-preparative high-performance liquid chromatography (semi-prep. HPLC) for mg amounts (Fig. 1b) (Chaaib et al. 2003; Mahmoud et al. 2020; Queiroz et al. 2002a; Weber et al. 2011; Zanolari et al. 2003). Compared to CC, the better control of pressure and LC conditions enables a rather rudimentary optimization of the separation with analytical high-performance liquid chromatography (HPLC).

Improving resolution at the preparative scale, is directly dependent on the stationary phase particle size and the solvent flow rate employed. MPLC and FC are operated with usually with 15–30 µm stationary phase particles (pressure: tens of bars) and semi- preparative HPLC with 5–10 µm particles (pressure: hundreds of bars) (Atanasov et al. 2015; Bucar et al. 2013; Sticher 2008). Semi-preparative HPLC has been mainly used for the final purification step of simplified fractions originating from the other preparative approaches (Angelis et al. 2020; Darme et al. 2022; Human et al. 2021; Jing et al. 2022; Keller et al. 2021; Zhou et al. 2022).

In parallel, at the analytical level, crude extracts were profiled with generic reversed-phase chromatographic gradients to tentatively assess their chemical composition. This capability was facilitated by the emergence of spectroscopic techniques coupled with HPLC (hyphenated techniques), a trend that gained momentum from the 1990s onwards (Holt et al. 1997; Jaroszewski 2005; Wolfender et al. 2001; 2003; Wolfender et al. 2005). These techniques involve the integration of analytical HPLC with detectors capable of providing real-time structural data, including photodiode array (PDA), mass spectrometry (MS), and even nuclear magnetic resonance (NMR) (Fig. 1b)(Queiroz et al. 2005, 2002b; Wolfender et al. 2005). Such approaches were mainly used to identify previously known NPs in extracts, while avoid their isolation, a process known as ‘dereplication’. This process was effective at the analytical level, but the multiple steps of the isolation methods used at the preparative scale did not facilitate the monitoring of known NPs and made the practical implementation rather complex. This process has not always been efficient enough to cope with the rapid turnaround and tight deadlines of modern high-throughput screening (HTS) programmes (Blay et al. 2020).

Today, NP investigations often involve advanced liquid chromatography–mass spectrometry (LC–MS) methods for in-depth metabolite profiling prior to the isolation work (Fig. 1c) (Wolfender et al. 2019). This involves analytical platforms combining ultra-high performance liquid chromatography (UHPLC) with sub-2 µm particles columns to achieve high-resolution, high-throughput chromatographic separations, together with a high-resolution mass spectrometer capable of data-driven acquisition to generate high-resolution mass spectrometry (HRMS) and tandem mass spectrometry (MS/MS) spectra for detailed compound annotation (Li and Gaquerel 2021; Perez de Souza et al. 2021; Wolfender et al. 2022). Such an approach provides putative structural annotation of most detected metabolites for automated early identification of known NPs (dereplication) and enables to rapidly estimate the composition of ENs prior to targeted isolation of given prioritized NPs (Fig. 1c)(Kind and Fiehn 2017). The introduction of such advanced metabolomics methods also enables massive metabolite annotation on large ENs collections. Mining of such data helps to highlight unusual/original compounds or given analogues that can be selected for targeted isolation procedures (Olivon et al. 2017, 2019, 2020; Quiros-Guerrero et al. 2022; Wolfender et al. 2019).

Similarly, in typical metabolomics investigations, metabolite profiling is used to compare a set of biological replicates for highlighting biomarkers through advanced multivariate analyses method (Wolfender et al. 2022). In both approaches, compound of interest can be precisely located in the analytical profiles of the corresponding natural extract (Wolfender et al. 2019). For an efficient isolation of such selected compounds, the preparative separation chromatogram should ideally be similar to the one obtained at the analytical scale. This implies to use HPLC preparative chromatographic methods with high selectivity and resolution, including an efficient transfer of the metabolite profiling conditions to allow effective targeted isolation of the LC peaks of interest (Fig. 1c).

For an efficient monitoring of the target molecules of interest in such cases, semi-preparative HPLC platform can be hyphenated with different detectors such as ultra-violet (UV), evaporative light scattering detector (ELSD), and mass spectrometry detection (MS).

This review article does not claim to be exhaustive on all methodologies developed and implemented by chemists for the isolation of NPs. Instead, the primary objective of this article is to emphasize recent studies that employ cutting-edge, high-resolution chromatographic methods at semi-preparative scales for NP isolation. In this context, chromatographic techniques operating at low resolution (CC, FC, MPLC) or based on liquid partitioning (CPC and HSCCC), although very useful for NP isolation, are not considered in this article and have been well discussed in other recent reviews (de Mello and Leitäo, 2023; Gong et al. 2020; Li et al. 2022; Yang et al. 2020; Zhang et al. 2023, 2018).

## Latest trends in preparative chromatography

As mentioned, today, the integration of metabolite profiling results for guiding isolation is an increasingly relevant aspect in NP research (Fig. 1c). Such analyses are carried out: (i) either for dereplication purposes, (ii) to anticipate structural novelty, (iii) as the result of HPLC-based activity profiling, (iv) or finally as the result of advanced metabolomics highlighting biomarkers to be identified. All these analyses provide the support for the precise definition of a HPLC peak or group of peaks of interest. Since the majority of current methods for profiling metabolites are performed using reversed-phase (RP) chromatography (Perez de Souza et al. 2021), the preparative methods discussed here will be related to this specific chromatography mode which is the most straightforward for MS detection.

Advanced LC–MS analytical platforms now permit exceptional chromatographic performances with the recent development of UHPLC (Perez de Souza et al. 2021; Wolfender et al. 2022). Likewise, due to the significant progress in MS, metabolites can now be detected over a broad dynamic range with unrivaled sensitivity. In metabolite profiling, it is usual to detect several hundreds of MS features in a natural extract (Bauermeister et al. 2022; Duhrkop et al. 2021; Li and Gaquerel 2021; Wolfender et al. 2022). So, the current challenge in ‘preparative methods’ (which we define here as all techniques enabling the isolation of a compound at any scale from micrograms to milligrams) will be to match, if not surpass, the efficiency of analytical chromatographic separation to ensure effective physical separation of the compounds. This requires knowledge of fundamental aspects of chromatography such as stationary phase morphology, stationary phase chemistry, and chromatographic modes (Dong 2019; Fanali et al. 2013; Snyder et al. 2010). In addition, it is important to link the monitoring of separations at the preparative scale with analytical profiling data to ensure proper follow-up for targeted isolation of prioritized NPs. It is, however, important to mention that the direct scale-up from UHPLC to preparative LC is not always straightforward. Indeed, the pressure generated by the column packed with sub-2 µm particles (UHPLC) is quite different from the one packed with 5 µm particles (semi/preparative HPLC), leading to possible frictional heating effects in UHPLC, which are responsible for changes in selectivity and retention. For this reason, in most of the studies dealing with natural products, people prefer to use an intermediate step of analytical HPLC to avoid such issue.

The main purpose of this section is to highlight and critically discuss all the major improvements that have been recently made: (i) to increase preparative chromatographic resolution (injection techniques, isolation directly at the analytical scale, recycle HPLC, 2D-LC), (ii) to determine the ways in which analytical profiling conditions can be precisely transferred to the preparative separation (chromatographic gradient transfer), and (iii) to optimize separation using software-based approaches (computer-assisted modelling in LC). The latest developments in coupling different detectors at the preparative level to guide fractionation will also be addressed (hyphenation to UV/PDA, ELSD, CAD or MS and NMR).

A critical view of the advantages and disadvantages of all these innovative preparative approaches will be developed. The compromises sometimes necessary for their implementation in the specific context of targeted NPs isolation will be discussed. Recent applications to the purification of ENs from different origins will illustrate these latest developments.

### Sample injection

In semi-preparative liquid chromatography, the most common way to inject a sample is to use a liquid injection with a loop injection valve. The main limitation is that the sample must be sufficiently soluble in a small volume of solvent. Ideally, the solvent used for sample solubilization should be similar or less eluent than the one used at the initial chromatographic conditions. However, in RP separation, at the start of the gradient, the mobile phase is mainly composed of water, in which the organic extract is often not completely soluble. The sample must therefore be dissolved in a pure organic solvent to avoid precipitation and potential blockage of injector needles, valves and capillaries. In addition, the use of a large volume of this solvent can have an impact on the quality of the separation for polar compounds. To overcome this limitation, modern HPLC instruments equipped with automatic injectors offer the possibility of "sandwich injection"(Agilent 2019). Using this approach, the sample can be embedded ('sandwiched') between two plugs of a suitable solvent that prevents precipitation in the sample loop (Agilent 2019). DMSO or water-immiscible solvents can be used.

**Fig. 2:**
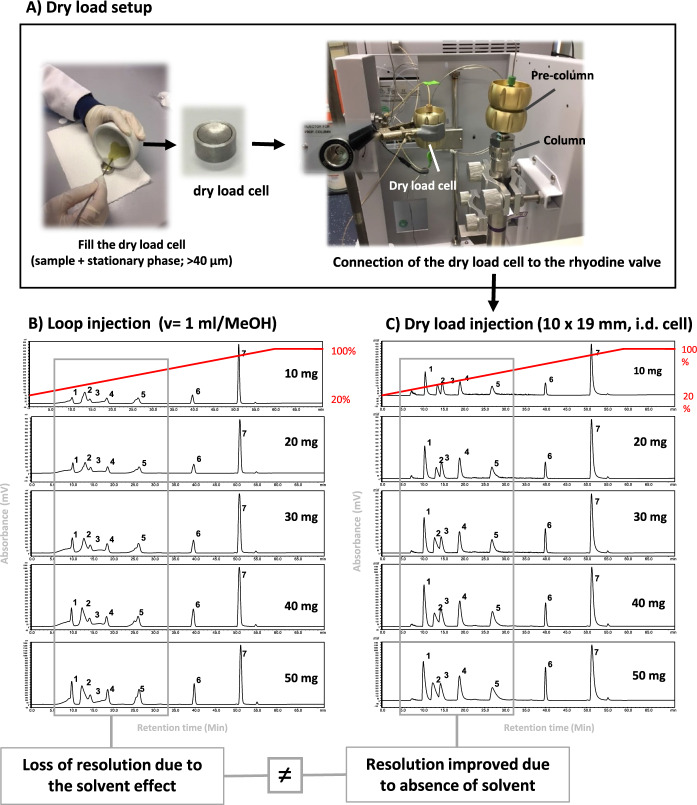
**a** Scheme illustrating the preparation of a dry load cell. Semi-preparative HPLC–UV separation of a standards mixture using the **b** loop injection (10 to 50 mg in 1 mL of MeOH), **c** dry load injection of 10 to 50 mg using a dry load cell of 10 × 19 mm, I.D. Elution order: caffeic acid (**1**), phenol (**2**), *p*-coumaric acid (**3**), rutin (**4**), quercetin (**5**), piperin (**6**), and anthracene (**7**). (Queiroz et al. 2019). This figure was adapted with permission from Queiroz EF et al., Journal of Chromatography A, 1598, 85-91. Copyright 2019 Elsevier

Alternatively, to eliminate the need for solvents, a new dry load technique has been developed for introducing samples into semi-preparative HPLC (Queiroz et al. 2019). This approach is based on the use of modified commercial pre-columns (10 × 19 mm I.D.) as dry load cells (Queiroz et al. 2019). For this purpose, the sample is mixed with the particles composing the stationary phase and introduced into the cell (Fig. 2a). The device has been connected to a Rheodyne® valve, which replaces the loop between the pump and the column inlet. This finally enables the chromatographic run to be initiated without the need for any injection solvent. This approach was validated with a mixture of representative NP standards where classical loop injection with methanol produces significant peak distortion and broadening (Fig. 2b), while the dry load injection affords symmetrical and narrow peaks (Fig. 2c).

This method has also been effectively used to introduce samples into flash chromatography (Azzollini et al. 2016), MPLC (Challal et al. 2015; Saldanha et al. 2021), and supercritical fluid chromatography, while maintaining resolution (Miller and Mahoney 2012), even with high sample amounts injected.

Another factor to take into account is the pre-treatment of the extract prior to purification. This matters because large quantities of lipophilic substances, such as lipids and pigments, or highly hydrophilic compounds like saccharides and tannins present in the extract, can significantly affect separation quality and lower the concentration of the target compounds (Ma et al. 2018). To remove these unwanted matrix components, methods like vacuum liquid chromatography (VLC) (Houriet et al. 2020; Queiroz et al. 2014; Saldanha et al. 2021), and liquid/liquid partition (LLP) (Alfattani et al. 2021, 2023; Klimenko et al. 2021, 2023; Pellissier et al. 2021) are typically employed. Applying such a treatment to the sample increases the concentration of the desired compounds in the sample, facilitating their efficient isolation.

In the last few years, dry loading has been successfully applied to the NP purification of both lipophilic and hydrophilic ENs (Klimenko et al. 2021; Neuenschwander et al. 2020; Queiroz et al. 2019; Righi et al. 2020; Saesong et al. 2019). However, one limitation of this type of sample introduction is that it is not yet fully integrated into a dedicated semi-preparative platforms. The process also requires precise sample preparation and training, and dry load cells are not commercially available.

Despite these restrictions, recent examples of applications of such sample introduction especially in conjunction with high resolution RP preparative methods demonstrate that all of these drawbacks are largely offset by the improvement in chromatographic resolution, leading to the better physical separation of the compounds (Ferreira Queiroz et al. 2023; Huber et al. 2022, 2023, 2021; Klimenko et al. 2021, 2023; Neuenschwander et al. 2020; Saesong et al. 2019).

### Gradient transfer from analytical to preparative scale for NPs isolation

One way to maintain comparable chromatographic profiles (equivalent selectivity and resolution) between the analytical and preparative scales is to apply the chromatographic gradient transfer. For this, some strict rules must be followed at the preparative scale. Firstly, the preparative LC column should be packed with the same packing chemistry as the analytical column, to avoid changes in retention and selectivity. If the same column chemistry is not available for larger-scale separations, it is possible to select an “equivalent” column from another source. To find out the most appropriate preparative column chemistry for a given application, software for comparing column selectivity for more than 500 columns is freely available on the US pharmacopeia website (USP 2021). The ratio of column length (L) to particle size (d_p_) must ideally remain constant to maintain comparable peak capacity and resolution between the two dimensions. The flow rate, injected volume, and gradient profile must be adjusted based on column dimensions, while dwell volume differences between the analytical and preparative LC instrument must be compensated by introducing an initial isocratic hold time at the start of the preparative gradient. All these calculations can be sometimes difficult to perform for inexperienced users, so an Excel tool to find out the geometrically equivalent conditions between analytical LC and preparative LC was developed (Guillarme 2020). Such a systematic method transfer procedure was successfully applied to isolate various NPs directly from complex extracts.

**Fig. 3:**
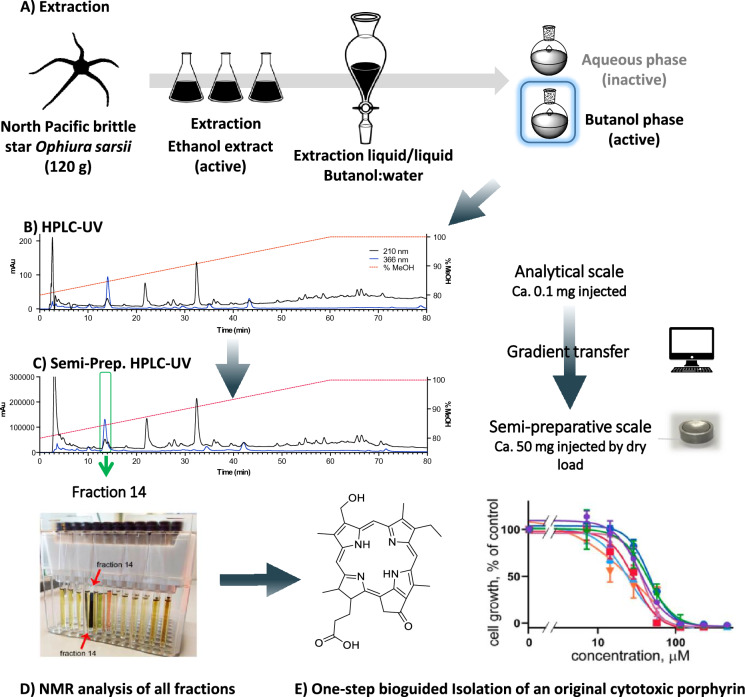
One step isolation of a bioactive chlorin derivative from the brittle star *Ophiura sarsii* (Klimenko et al. 2021). **a** Increasing polarity extraction followed by elimination of saccharides and inorganic salts by liquid/liquid fractionation **b** Optimization of the separation at the analytical scale of the active butanolic phase **c** Gradient transfer of the conditions to semi-prep HPLC–UV. **d** NMR analysis of fractions obtained. **e** structure of the original porfhyrin isolated and cell proliferation (MTT) assay shows a broad cytotoxicity of the compound against breast cancer lines. This figure was adapted with permission from Klimenko A et al., Marine Drugs, 19(1), 11; 10.3390/md19010011 (2021)

For example, Klimenko et al*.* (2021) have demonstrated a one-step preparative scale isolation of a complex cytotoxic porphyrin from the North Pacific Brittle Star *Ophiura sarsii*. In this case, saccharides and inorganic salts were eliminated from the methanolic extract using LLP with butanol and water. The butanolic enriched fraction obtained was analysed by HPLC–UV. The combination of gradient transfer and dry load injection allows the isolation of the compound of interest in one single step (Queiroz et al. 2019) (Fig. 3). Recently, this marine organism has been the subject of a second chemical study where molecular network analysis has allowed the localisation the minor analogues of the first chlorine isolated of this species. A targeted isolation using the described methodology allowed the isolation of five additional chlorin derivatives in a single step (Klimenko et al. 2023).

**Fig. 4:**
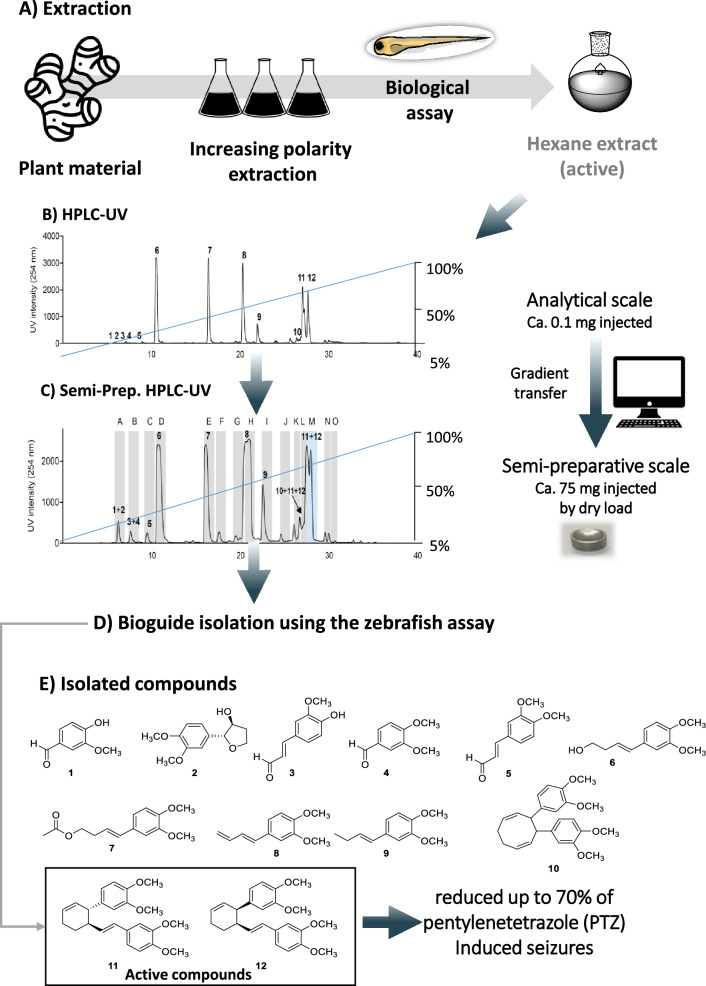
Bioguided isolation of the active compounds from the hexane extract of *Zingiber purpureum*. **a** Plant extraction using increase polarity extraction with hexane, dichloromethane, and methanol. Selection of the hexane extract based on the biological assay using zebrafish larvae (Afrikanova et al. 2013). **b** Reverse phase HPLC–UV analysis of the hexane extract. **c** Gradient transfer of the conditions to the reverse phase semi-preparative HPLC–UV and sample injection by dryload. **d** Biological assay using the zebrafish larvae. **e** Structure of the isolated compounds, 11 and 12 of which were identified as the active principles. This figure was adapted with permission from Brillatz et al., Journal of Agricultural Food and Chemistry, 68, 7904-7915. Copyright 2020 American Chemical Society. (2020)

In another example, the chemical content of the rhizomes of *Zingiber purpureum*, was investigated for its antiseizure properties using a streamlined and cost-effective zebrafish screening strategy (Brillatz et al. 2020). Its hexane extract demonstrated strong antiseizure activity in a zebrafish epilepsy assay (Afrikanova et al. 2013), and was, therefore, selected for bioactivity-guided fractionation. The extract was analysed by analytical HPLC–PDA using a reverse phase stationary phase (Fig. 4). These conditions were successfully transferred to the semi-preparative HPLC–UV scale, and 75 mg of the extract was purified (Fig. 4). Despite the column overloading and thanks to the use of a dryload injection (Queiroz et al. 2019), the separation remains precise, and it was possible to keep a good resolution between most peaks. Using this approach twelve compounds were isolated including two bioactive phenylbutenoids, *trans*- (11) and *cis*-banglene (12). These compounds reduced up to 70% of pentylenetetrazole (PTZ)-induced seizures (Fig. 4) (Brillatz et al. 2020).

As shown, chromatographic gradient transfer facilitates the alignment of LC peaks of interest between analytical and preparative scales, which is particularly useful when dealing with complex mixtures. However, some of its possible drawbacks include the need for thorough platform characterization, particularly system dwell volume and column dead volume, to achieve transposable separation at the preparative scale. As we have already explained, it is essential to use the same stationary phase on both the analytical and preparative scales, which is not always possible. However, compromises can be found by using closely related stationary phases. When using common detection at both analytical and preparative scales, compounds of interest can also be identified by comparing LC peak patterns in a chromatography region of interest.

### Ultra-high pressure liquid chromatography for NPs isolation at µg scale

Despite the aforementioned advances, the presence of closely related structural analogues in extracts represents a challenge to obtain high-purity NPs and in certain cases chemically similar NPs cannot be effectively separated even with preparative high-resolution techniques. Although the structures of these compounds can be determined in mixture by NMR (Gruene et al. 2018; Kunde and Schmidt 2019), the evaluation of biological activity requires a high degree of purity (Choules et al. 2018).

**Fig. 5:**
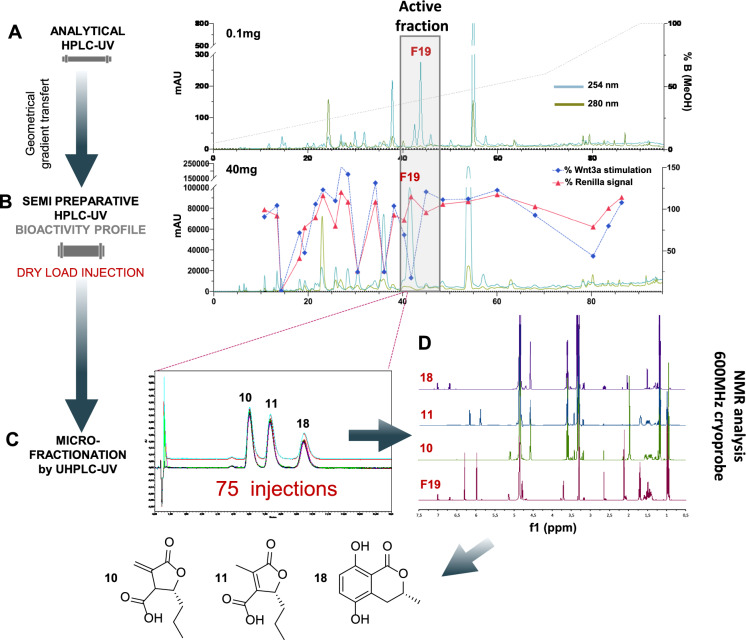
Isolation of minor bioactive compounds from the endophytic fungus *Lasiodiplodia venezuelensis*. **a** Analytical HPLC–UV profile of the enriched hydroalcoholic fraction of *L. venezuelensis* extract. **b** Semi-preparative HPLC–UV profile of the hydroalcoholic fraction with an overlay of the Wnt inhibition bioactivity profile of the fractions. The fraction named F19 was the only one to show specific anti-Wnt activity, while other fractions were cytotoxic and the decrease of Wnt signal was due to this effect. **c** UHPLC-PDA purification of F19 repeated 75 times on a phenyl column. **d** Comparison of the ^1^H-NMR spectra of F19 and the isolated compounds 10, 11, and 18. This figure was adapted from Pellissier L, et al. (2021) Frontiers in Microbiology, doi: 10.3389/fchem.2021.664489 (2021)

Recently, UHPLC has demonstrated its efficiency to purify closely related compounds with selective Wnt (wingless/integration 1) inhibition from a mixture that cannot be separated by conventional semi-preparative HPLC–UV (Fig. 5a). In this approach, the enriched hydroalcoholic fraction of an endophytic fungus *Lasiodiplodia venezuelensis* was first purified by RP semi-preparative HPLC using a 5 µm high resolution reverse phase stationary phase (Fig. 5b)(Pellissier et al. 2021). From this experiment, 15 compounds were successfully purified in a single step, however, the biological activity was concentrated in a fraction still containing a mixture of 3 compounds. To achieve sufficient resolution, these compounds were further purified by UHPLC-UV on a 1.7 µm column packed with phenyl stationary phase (Pellissier et al. 2021). Since the loading capacity of these columns is very limited, 75 injections of 8 µg each were required (600 µg of the fraction injected) to obtain sufficient amount of pure compounds (Fig. 5c). The high reproducibility of UHPLC enabled an efficient repeated collection of all peaks at the microgram scale (Fig. 5c).

The small fraction volumes corresponding to the three peaks were collected thanks to an analytical UHPLC fraction collector yielding an efficient isolation of these compounds at the µg scale (Pellissier et al. 2021). The minute amount obtained was however sufficient for structural elucidation by sensitive NMR and biological activity assessment. Such an approach demonstrates that the isolation and subsequent full de novo characterisation of bioactive NPs is possible at the microgram scale (Fig. 5d). A similar approach was recently applied to isolate a damage-response jasmonate precursor in the *Arabidopsis thaliana* leaf vasculature. UHPLC fractionation was the only valuable alternative due to the very small amount of leaf vein tissues available (Morin et al. 2023).

Application of analytical UHPLC for NP isolation demonstrates exceptional precision and reproducibility. Importantly, there is no need to transfer the method to a different scale. It achieves unmatched resolution by utilizing the smallest particle stationary phase available on the market. Moreover, at this analytical column scale, a wide array of stationary phase chemistries is accessible. This technique has, however, limitations related to the low amount that can be injected onto the column, requiring a non-negligible number of injections. In addition, great care in sample preparation is mandatory due to the small porosity of the inlet/outlet frits. This approach is limited to the purification of fractions obtained by a first preparative chromatographic step. There is also the need for sufficiently sensitive NMR and bioassays to characterize the targeted NPs at the microgram scale.

### Recycling HPLC to maximize efficiency

**Fig. 6:**
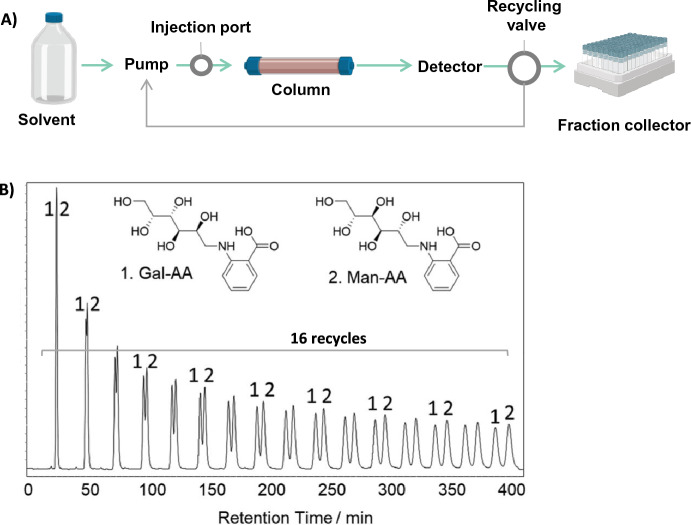
Purification of two natural products using recycling HPLC. **a** Closed-loop recycle HPLC set-up. Separation of purified natural glycans (monosaccharide-AA conjugate mixtures): **a** Gal-AA(**1**)/Man-AA. (**2**); **b** Gal-AA(**1**)/Man-AA(**2**)/Glc-AA(**3**). Column: Luna C18 (21.2 × 250 mm); Mobile phase: 8% acetonitrile/0.1% TFA; Flow rate: 12 mL/min. Injection amount: 1 mg/mL. This figure was adapted with permission from Zhu Y. et al., Analytical Chemistry, 599, 113702. Copyright 2020 Elsevier. (2020)

When separating isomeric or closely related NPs, the chromatographic resolution can be improved by simply extending the column length (increase of the plate number). However, this solution is not always adapted, as very long columns are not commercially available, and the serial coupling of several columns can be an expensive solution, which also dramatically increase the pressure drop. In this context, recycling HPLC can be a good option (Gritti et al. 2018). In this technique, by using connected valves, the sample (selected unresolved peak of interest) will pass several times on the same column (Fig. 6a). A recycle valve is positioned post-column in the HPLC system, to recirculate several times unresolved peaks in the column, without increasing the system pressure. This approach has been recently applied for the purification of a plethora of NPs (Bihud et al. 2019; Huber et al. 2023; Hussain et al. 2020; Jibril et al. 2019; Moreno-Velasco et al. 2022; Suarez-Ortiz et al. 2017). Recently, Song et al*.* used a closed loop recycling HPLC at the preparative scale to purify natural glycans. The mixture of compounds had to be recycled 16 times through the column to achieve sufficient resolution (Fig. 6b) (Zhu et al. 2020).

Recycling HPLC is clearly restricted to the purification of selected fractions and modern platforms offer a high degree of automation for such operation. However, its use should be based on the balance between the benefits (higher peak purity) and the challenges associated with the complexity of the setup. Implementing recycling HPLC requires additional hardware. In addition, there is a high risk of carryover, as a portion of the previous injection is reintroduced into subsequent injections. This can result in contamination of subsequent samples. Finally, developing a recycling HPLC method can be more challenging than conventional HPLC. Indeed, the cycling parameters (i.e. number of cycles, injection volumes, and recycling flow rates) need to be carefully optimized.

### Multidimensional preparative LC to maximize selectivity

Another way to improve separation efficiency is to combine separation on columns with complementary selectivities. In the case of NP isolation, this operation is typically performed on the fractions from the first separation, which are then purified on a column with a different stationary phase to that used for the first coarse fractionation.

In a forward-looking perspective, the development of automated 2D-LC approaches on a preparative scale needs to be addressed. This strategy is particularly attractive for the separation of complex mixtures such as ENs, because it offers a potential improvement in terms of peak capacity, selectivity, and resolution (Pirok et al. 2019). From a commercial point of view, efficient multidimensional LC instruments are now available such as Sepbox® (Bhandari et al. 2011), and Sepiatec® (Lubanyana et al. 2020), affording the possibility to automatically perform multidimensional separation of complex extracts using different stationary phase chemistries, thus generating fractions with lower complexity, and also in some cases, pure compounds (Coulerie et al. 2016). For these instruments, an automated optimized protocol is used. It is based on the implementation of orthogonal separations on different reverse-phase columns, and the conditions are adapted to the compound polarity. Between the two chromatographic dimensions, NPs are trapped onto several solid phase extraction (SPE) cartridges and then reinjected into the second dimension. As for the dry load introduction presented in the section dedicated to "Sample injection", this trapping guarantees a better resolution of the second-dimension separation and less compromise has to be made in terms of solvent compatibility. On such platform, the 2D separation typically generates 100–200 fractions that are automatically collected for a given extract (Coulerie et al. 2016). In the field of drug discovery, such platforms can be used as for the automatic generation of fractions and pure compounds from biological screening (Coulerie et al. 2016). They can also be used for compound purification by combining a normal phase separation in the first dimension with a reverse phase separation in the second one (Li et al. 2016; Wang et al. 2015).

The sepbox® was for example successfully used to purify a series of saponins from 4.9 g of a methanolic extract of *Panax notoginseng* (Lelu et al. 2016). For this, separation was performed on a first C4 column using water-acetonitrile gradient. Fractions were trapped on 15 SPE cartridges and subsequently separated using different gradients of water, methanol and acetonitrile on a C18 column generating a total of 116 individual subfractions containing either pure compounds or very simplified fractions. Subfractions were further purified by semi-preparative HPLC. Using this approach, 11 compounds were purified and identified (Lelu et al. 2016).

Developing an effective 2D-LC method can be challenging due to the need to optimise several separation dimensions and ensure compatibility between them. Thus despite the significant gain in resolution achievable with multidimensional LC, it is important to consider that 2D-LC preparative systems are more complex to set up and operate than traditional one-dimensional systems. In addition, 2D-LC preparative systems often require the use of multiple detectors, columns, and pumps, which can result in higher equipment costs compared to simpler chromatographic setups. Nevertheless, thanks to the high level of automation and reproducibility reached on modern platforms, it is possible to obtain series of pure compounds in a short time and/or generate libraries of fractions for screening purposes (Chen et al. 2020; Lelu et al. 2016; Wang et al. 2019).

### Computer-assisted modelling in LC for targeted NP isolation

Although the improvement in chromatographic resolution and selectivity on modern platforms has been significant over the years, one of the key features to consider is the optimization of mobile phase and gradient conditions. To this end, computational methods have been developed to enable efficient experimental design and streamline separation optimization steps on both analytical and preparative scales. As this is a technique that requires fundamental knowledge of chromatography to understand its potential, the following section provides an introduction to computer-assisted modelling in HPLC.

#### Introduction to computer-assisted method development

In LC, retention, and selectivity can be optimized using a simple trial and error approach based on the variation of one factor at a time, including stationary phase chemistry, organic solvent nature, pH, temperature, isocratic composition, and gradient conditions. Such method development process is often tedious and time-consuming because of the high probability of peak overlap in complex mixtures and the dependence of retention and selectivity on the chromatographic parameters. As an alternative, “computer-assisted method development”. facilitates method development. Such software can predict separation as a function of one or more experimental conditions, after having performed only a limited number of preliminary experiments. The possibility to generate simulated chromatograms strongly reduces the amount of experimental work, as there is no need to carry out numerous “real” experiments. Several modelling software have been developed in the last 25 years for LC, including Drylab®(Molnar 2021), Chromsword® (Chromsword Solutions), ADC/Autochrom®(ACD/Labs 2021), and Osiris® (Interchim 2021). Drylab® is considered the most comprehensive and widely used computer simulation software presently available (Molnar 2021). Today, none of the software listed here is open source, and all of them require a license which can be quite expensive.

**Fig. 7:**
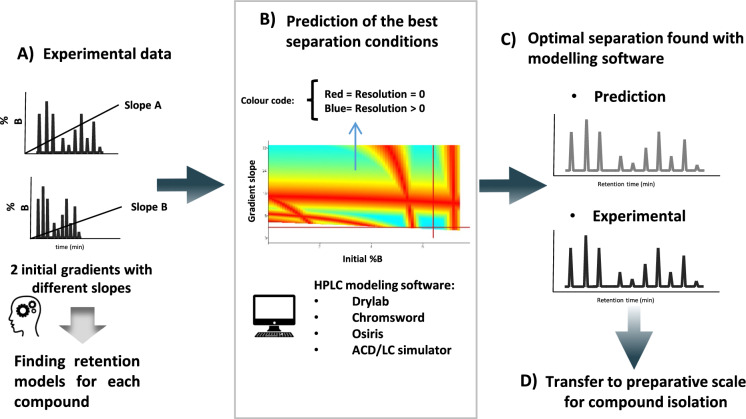
LC optimization via computer-assisted modelling for the separation of complex mixtures. **a** Experimental data consisting in the analysis of a complex mixture by two different gradients conditions. The retention models of each compound can then be defined with the modelling software. **b** Resolution map provided by the modelling software and showing the evolution of minimal resolution vs. gradient conditions. **c** Comparison of the experimental and predicted separations. **d** Transfer of analytical conditions to the preparative scale for compound isolation

All these software packages allow measuring and visualizing the effect of mobile phase conditions (gradient time and profile, pH, ionic strength, additive concentrations, temperature…) for a given stationary phase. As described elsewhere (Mondello 2019), computer simulation makes use of various empirical and theoretical fundamental equations of chromatography to predict retention times and peak widths for either isocratic or gradient elution. The most useful information provided by computer simulation is a resolution map (Fig. 7), which is a plot of the critical resolution Rs, for the two least-resolved peaks (or two selected peaks of interest in the chromatogram), as a function of the conditions that were varied in the initial experimental runs. When using modelling software, an experimental design should be first selected, which defines the number of initial experiments that are required for computer simulation to model the evolution of selectivity (resolution) as a function of mobile phase conditions on a selected column. For example, only two gradient experiments differing in time are required to model a few thousand experiments with an accuracy of around 99% and find out the optimal gradient conditions (Fig. 7). Four experiments are required if gradient conditions must be optimized together with mobile phase temperature (two gradient times at two temperatures). Six experiments are needed to simultaneously optimize gradient conditions and pH conditions (two gradients at three pH in a relatively narrow pH range of maximum 3 units). Finally, thanks to the development of modelling software, three-dimensional (3D) resolution cubes can also be built based on 12 experiments to simultaneously optimize three parameters at the same time, such as gradient conditions, pH, and temperature.

The value of computer simulation is particularly high when one or more of the following conditions applies: (i) separation is performed by gradient elution, (ii) complex samples containing at least 5–10 components, or with a lot of closely eluted peaks, (iii) short runtime is required.

#### Computer-assisted retention modelling at the preparative scale

**Fig. 8:**
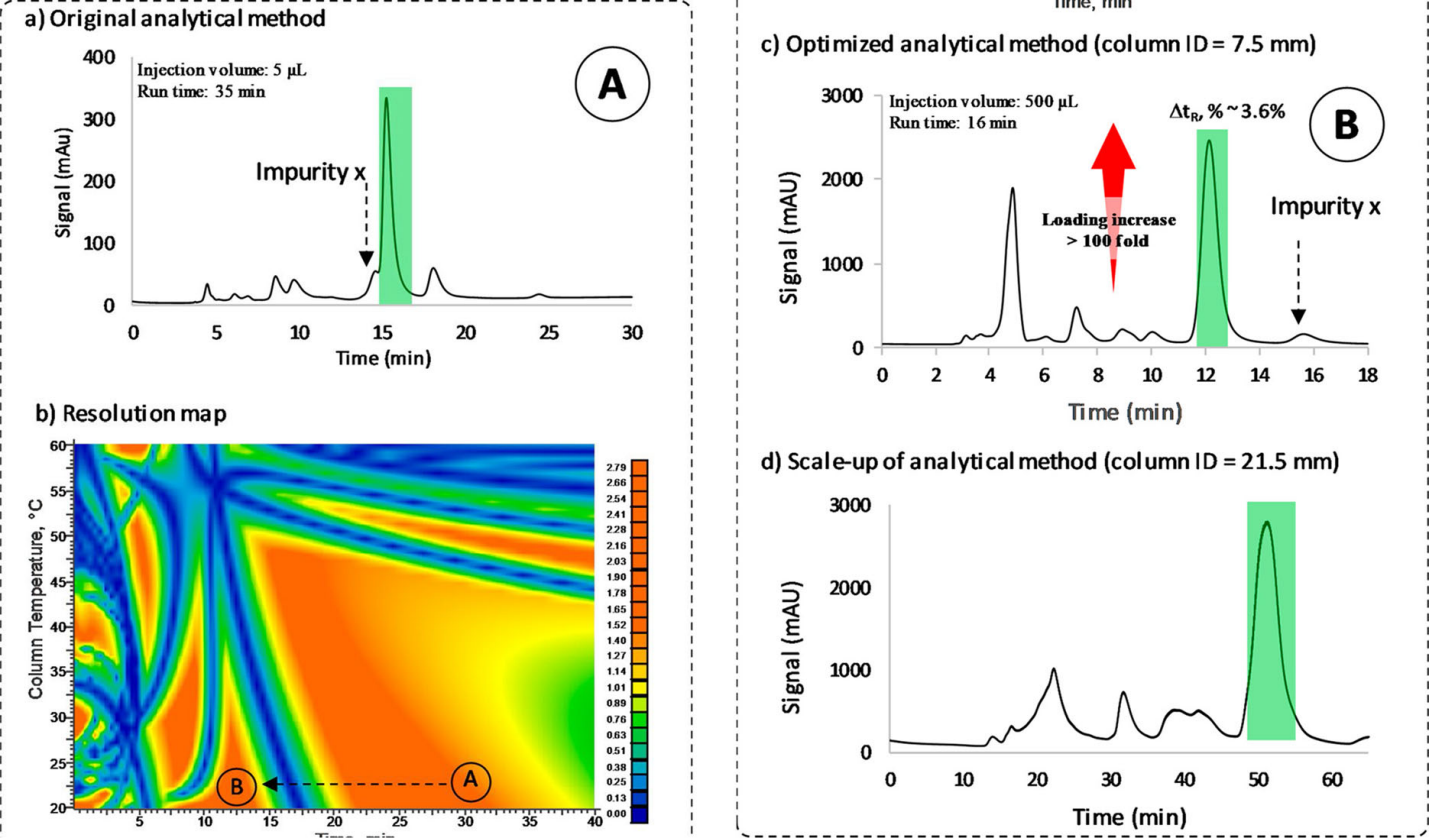
Separation of a beta lactam derivative using multidimensional preparative HPLC. **a** Primary analytical HPLC method. **b** Resolution map generated from experiments at different gradient times and column temperatures. **c** The analytical method was obtained from the optimization of gradient time. **d** The method applied to purification of target component The resolution factors (Rs) were estimated as described in the Experimental Section. Relative standard deviation (RSD) < 4% (*t*_R,exp_ vs. *t*_R,pred_). This figure was adapted with permission from Ahmad IAH et al, Anal. Chem., 2020, 92, 13443-13451. Copyright 2020 American Chemical Society (2020)

The interest in computer-assisted method development at the preparative scale has been demonstrated in several articles, to make the up-scaling process easier and faster, as illustrated in Fig. 8. Wennberg et al*. *(2001) were among the first ones to demonstrate the added value of modeling software at the preparative scale in the field of plant analysis. A test mixture of six phenolic compounds was first analysed at the analytical scale and the method was fully optimized using two different gradient runs and Drylab®. The final run suggested by the software matched well with expected elution times and this upscaling procedure was finally successfully applied to a plant extract. Modeling software (Osiris®) was effectively used at the semi-preparative scale by Glauser et al*. *(2008) for the isolation and full de novo structure determination of minor-key plant biomarkers at the µg scale. The purification strategy relied on the optimization of the chromatographic analysis under RPLC analytical conditions transferred to semi-preparative RPLC (Glauser et al. 2008). Adili et al*. *(2020) described the development of capacity orthogonal chromatography (COC), a new technique for simultaneously determining the loading capacity and orthogonality during the construction of two-dimensional (2D) separations, based on the use of modelling software (Drylab®). The developed approach was used to successfully purify low-abundance corilagin from pomegranate flower extract.

Ahmad et al. (2020) have also recently demonstrated that computer-assisted chromatographic modelling allows gaining massive productivity increases (shorter cycle time and higher sample loading) for purification of pharmaceuticals by selectively switching the elution order of target components away from undesired tailing peaks and co-elution spaces . The authors applied this concept for the purification of a beta lactam antibiotic from a complex mixture using an ion exchange chromatography with a TOSOH TSKgel DEAE-3SW column (75 × 7.5 mm, I.D.) packed with a 10 μm strong anion exchange resin. A resolution map was generated using a 4 × 3 factorial designs based on different column temperatures (20, 30, and 40 °C) and mobile phase composition (0–100% B for 10, 20, 30, and 40 min) on the same column (Fig. 8). After mapping the separation landscape of the mixture, new conditions were quickly generated using the multifactorial peak crossover concept (A—> B) with changing mobile phase gradients. The undesired byproduct's elution order on the front of the target active pharmaceutical ingredient (API) peak was switched to its tail, as shown in Fig. 8, resulting in a 100-fold increase of sample loading, from a 5 μL injection at 10 mg/mL mixture to 500 μL of the same concentration (Ahmad et al. 2020).

Although retention modelling is highly effective for optimizing separation, it is not yet widely used in NP research. The main limitation to its wider acceptance is the complexity of the modelling software, which can be a barrier for beginners and requires additional training and time investment. Advanced software tools can have a non-negligible cost, which could also be a drawback for small laboratories or researchers with limited budgets. Finally, it is not always easy to obtain valid models, due to the need to accurately measure system dwell volume and column dead time, and considering the fact that retention models are not always linear. Last, the retention prediction in SFC (Tyteca et al. 2015) or HILIC (Tyteca et al. 2014) is much worse than in reverse-phase liquid chromatography (RPLC) or normal phase liquid chromatography (NPLC), thus limiting the applicability of modelling software in these two modes of chromatography.

### Integration of multiple detection (UV, ELSD, and MS) for targeted NP isolation

As explained above, the selection of NPs for isolation in drug discovery or metabolomic studies is currently mainly based on metabolite profiling data using MS detection. For targeted isolation, it is therefore important to have preparative scale detection methods that provide effective monitoring directly comparable to the metabolite profiling chromatograms.

At the analytical level, today most extracts are analyzed using platforms equipped with several detectors such as PDA-UV, ELSD, and MS, better evaluation of peak purity (Swartz 2010) and it is important to have similar detectors available at the semi-preparative scale.

UV detection is by far the most widely used to control preparative isolation and short optical pathlength UV flow cells prevent signal saturation while they can work at multiple wavelengths simultaneously (Agilent 2019; Shimadzu 2021). UV detection depends on the compound chromophore, and this can lead to errors in estimating the relative quantities of metabolites to isolate and/or neighboring interfering peaks in mixtures.

In this context, ELSD can be an alternative for detecting compounds transparent to UV, and offers universal detection for most NPs, except highly volatile ones (Ganzera and Stuppner 2005; Magnusson et al. 2015; Megoulas and Koupparis 2005). ELSD provides for most NPs unbiased quantitative detection while enabling a more reliable peak collection. As ELSD is a destructive method, it requires an efficient splitting device between the detector and the fraction collector (Agilent 2019; Shimadzu 2021). Semi-preparative HPLC coupled to ELSD was successfully used for the purification of various NPs with weak or no chromophores such as lipids of the eggs of the insect *Pieris brassicae* (Stahl et al. 2020), steroid saponins in commercial extracts of *Yucca schidigera* (Sastre et al. 2017), oligosaccharides (Munkel and Wefers 2019), and triterpenes (Ding et al. 2019).

MS detection at the preparative scale enables a direct precise monitoring of metabolites highlighted by metabolite profiling of extracts using the retention time and *m/z* data of the features of interest. MS offers high sensitivity and can automatically trigger collection of targeted compounds via the specific detection of the corresponding *m/z* (Agilent 2019; Shimadzu 2021) Implementation of MS at the preparative scale requires efficient splitting to avoid detector saturation (Azzollini et al. 2016). Detection involves generally a single quadrupole mass spectrometer with an electrospray ionization (ESI) source for separation using RP stationary phase (Agilent 2019; Shimadzu 2021). Alternatively, apolar compounds can be isolated via NPLC using an atmospheric pressure chemical ionization (APCI) source (Agilent 2019; Kagan et al. 2004; Shimadzu 2021). The application of MS detection at the preparative level is still scarce but recent applications in NP research demonstrate its potential. RP Semi-preparative HPLC-ESI/MS has been for example successfully used to purify ellagitannin from a concentrated tannin extract of *Punica granatun* (Barbieri and Heard 2019), and flavonoids from the hydroalcoholic extract of the aerial parts of *Croton gratissimus* (Pudumo et al. 2018).

**Fig. 9:**
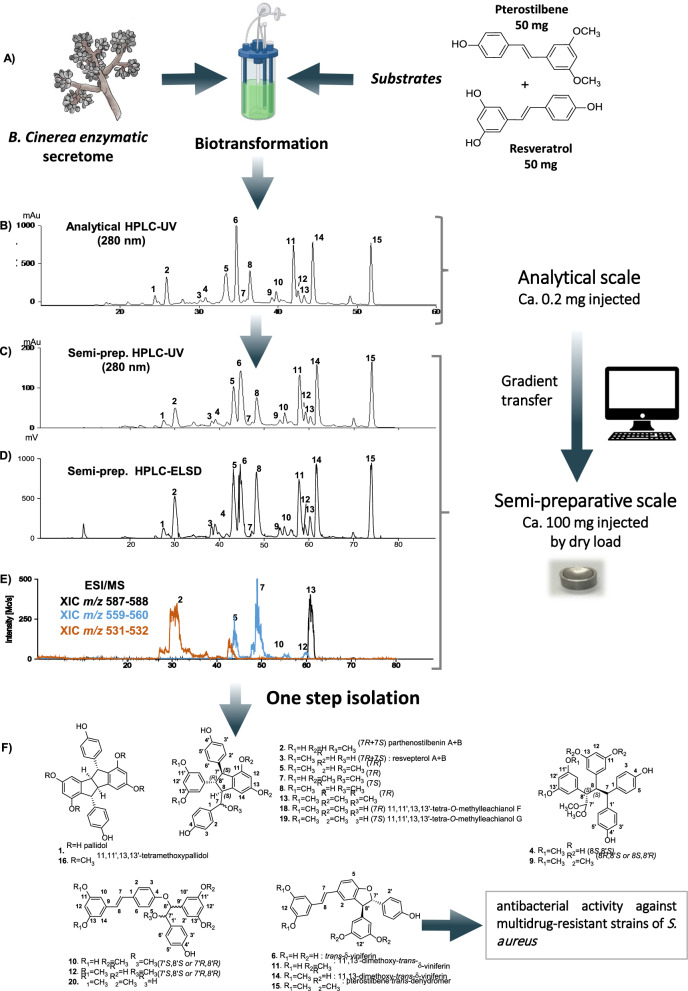
Integration of multiple detection for targeted NP isolation. **a** Biotransformation reaction of resveratrol and pterostilbene with the secretome of *B. cinerea* **b** HPLC–UV analysis at the analytical scale. **c** and **d** HPLC–UV-ELSD-MS at the semi-preparative scale where the sample was injected by dryload injection. **e** Extracted-ion chromatogram (XIC-MS) traces recorded by MS during the semi-preparative separation. **f** Structure of the isolated compounds. This figure was adapted with permission from Righi D. et al., Journal of Natural Products, 83, 2347-2356. Copyright 2020 American Chemical Society (2020)

Righi et al*. *(2020) have reported on a semi-preparative HPLC–UV-ELSD-ESI/MS multi-detection system for purifying a complex mixture obtained by biotransformation. Metabolite profiling with UHPLC-HRMS highlighted the presence of original compounds when the biotransformation reactions were performed with organic solvents (Righi et al. 2020). UV, ELSD, and MS monitoring afforded complementary information on the separated compounds. The UV trace matched the signals of the ELSD trace, which can be explained by the almost identical chromophore of all the compounds (Fig. 9c, d). The extracted ion chromatogram (XIC-MS) detection trace enabled a specific monitoring of the minor compounds (e.g., 7 and 13) for their targeted fractionation (Fig. 9e). This advanced purification procedure, applied on 50 mg of the biotransformation reaction, yielded 21 pure compounds in a single step (Righi et al. 2020) (Fig. 9f).

A normal phase preparative MS-guided isolation approach was used to target the isolation of the antifungal compounds from the Chinese liverwort *Chiloscyphus polyanthos* (Azzollini et al. 2016), 3.5 g of a dichloromethane (DCM) fraction was purified using a high-resolution flash chromatographic (HRFC)-UV system hyphenated to an APCIMS detector. Using this approach, seven diterpenes were isolated, some of them being responsible for the antifungal activity observed in the plant extract (Azzollini et al. 2016).

Multiple detection at the preparative scale provides a precise view of the target compounds and matrix interferents. It is therefore very useful when purifying complex extracts and fractions. ELSD detection offers the possibility to track the isolation of non-active UV compounds, while MS enables precise targeted isolation by triggering the collection of selected ions. However, hyphenation of MS with preparative HPLC is difficult due to the detector's susceptibility to contamination from concentrated analytes or matrix interferents. In addition, MS and ELSD detectors must use dedicated split systems to avoid overloading. Finding the correct compromise for adjusting the correct eluent splitting between all detectors remains a challenge at the preparative scale. Finally, the implementation of such a multiple detection platform at the preparative scale is expensive and involves high maintenance costs.

A possible alternative to direct MS monitoring at the preparative scale is the LC–MS analysis of selected fractions for post-separation localization of analytes of interest. Such approach is effective if the preparative separation is perfectly aligned with the analytical conditions determined by gradient transfer as highlighted in the Sect. (2.2).

### At-line hyphenation of NMR with semi-preparative HPLC

As mentioned above, modern MS-based metabolite profiling approaches combined with molecular networks represent effective tools for the dereplication of NPs in extracts (Wolfender et al. 2019). MS annotation however requires additional spectroscopic data to ascertain structural identification. Furthermore, MS detection alone does not provide an unbiased semi-quantitative estimation of a given extract composition. To obtain such complementary information nuclear magnetic resonance (NMR) is of prime importance.

To this end, the coupling of NMR with HPLC (LC-NMR) has been successfully employed. However, this requires a number of compromises (peak eluting in large elution volume, solvent suppression of the organic modifier, …), and only major compounds can be detected by 1D ^1^H-NMR (Wolfender et al. 2006).

An effective alternative is the trapping of analytes of interest post column on solid phase extraction (SPE) cartridges. This approach at the analytical level has led to the development of LC-SPE-NMR, which has proved highly effective for NP identification. It can be considered as an automated isolation method, since the LC peaks of interest are physically separated and trapped prior to NMR analysis. Spectra are obtained after desorption of the cartridges with a deuterated solvent, either on-line (NMR flow cell) or off-line (NMR tubes)(Jaroszewski 2005). As NMR detection is non-destructive, the recovered micro-fractions can also be used for bioactivity assays. Recently, advanced applications have enabled the rapid identification of bioactive compounds of different kinds directly in crude extracts (Exarchou et al. 2015; Kalala et al. 2020; Liang et al. 2021; Pedersen et al. 2020; Petersen et al. 2019; Sturm et al. 2021).

Liang et al. described the use of HPLC-SPE-NMR to study the ethyl acetate extract of the Chinese medicinal herb *Rhododendron capitatum*, which showed interesting α-glucosidase and/or PTP1B inhibitory activities (Liang et al. 2021). In order to locate the constituents correlated with these biological activities, the extract was subjected a HPLC microfractionations on 96-well microplates. The fractions from each plate were subjected to the biological assays, allowing effective correlation of the biological activities to certain peaks on the HPLC–UV chromatograms. To identify these active HPLC peaks, the crude extract was first separated into four SPE fractions to reduce the complexity of the mixture and increase the yields of the compounds to be analysed. In a second step, the SPE fractions were subsequently analysed by HPLC–UV-HRMS-SPE-NMR. The peaks of interest were trapped on SPE cartridges after 10 to 15 consecutive separations depending on the sample (300 to 400 µg per injection).

This approach has yielded a series of original chromene merotenoids and capitachromene acids isolated in the low milligram to the microgram range, 5 of which are responsible for the biological activities recorded in the crude extract (Liang et al. 2021).

**Fig. 10:**
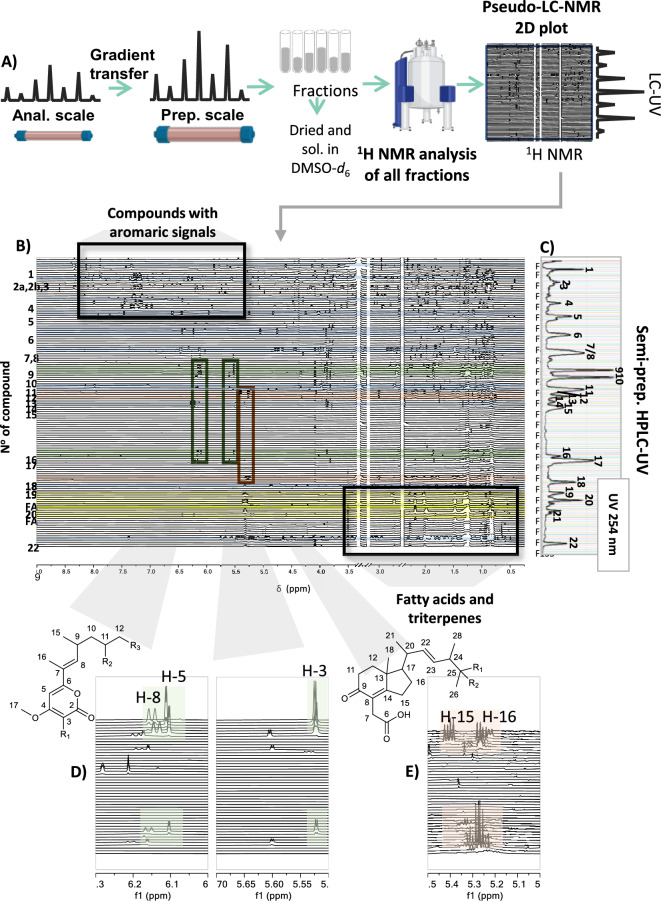
Pseudo-LC-NMR analysis of the ethyl acetate extract of *Fusarium petroliphilum*. **A**) Analytical HPLC conditions were transferred to the semi-preparative HPLC. All fractions obtained by semi-preparative HPLC were dried and submitted to the ^1^H-NMR analysis using DMSO-*d*_6_ as a bridging solvent. NMR data were combined in a pseudo-LC-NMR 2D plot. **B**) Stacked view of the ^1^H-NMR spectra of all fractions ordered by increasing retention time from the top to the bottom. Inserts showing the characteristic ^1^H-NMR signals of aromatic compounds, fatty acids, and triterpenes. (**C**) UV trace of the semi-preparative HPLC chromatogram for LC peak localization. (**D** and **E**) Inserts showing the characteristic ^1^H-NMR signals of some compounds on a set of fractions. This figure was adapted from Alfattani A, et al. (2021) Frontiers in Molecular Bioscience, doi:10.3389/fmolb.2021.725691

In HPLC-SPE-NMR, however, the use of analytical columns limits sample loading and often repeated injection of extracts for multiple trapping of the analyte of interest is required. To avoid multiple injection, high resolution semi-preparative HPLC represents an interesting alternative. It has been demonstrated that in a single separation step, it is possible to obtain a sufficient amount of compounds from a crude extract to record 1D and 2D NMR spectra on a sensitive cryogenated NMR platform. This approach was recently used for the isolation of antibacterial compounds from *Fusarium petroliphilum* an endophytic fungal strain isolated from a seagrass endemic to the Mediterranean Sea (*Posidonia oceanica*) (Alfattani et al. 2021). The ethyl acetate extract of that strain exhibited antimicrobial activities against *Staphylococcus aureus*. The identification of its active metabolites was performed in two steps. First, the extract was analysed by UHPLC-HRMS/MS molecular networking for dereplication and peak annotation. On the other hand, a single-step high-resolution fractionation was performed using semi-preparative HPLC–UV with a similar gradient profile. The fractions obtained were analysed by ^1^H-NMR and the data assembled into a 2D contour map, called “pseudo-LC-NMR” (Fig. 10). This approach facilitated the localization of structurally related compounds and provides an unbiased quantitative analysis of the constituents of the extract.

This innovative strategy led to the unambiguous characterization of 22 compounds (Alfattani et al. 2021). Among them, six inhibitors of the quorum sensing mechanism of *S. aureus* and *Pseudomonas aeruginosa* were effectively identified. Minor secondary metabolites were also characterized with good confidence by propagation of the annotation through the corresponding HRMS/MS molecular network, which enabled a consistent annotation of 27 additional metabolites.

Recently, a methodology based on trapping-enrichment deuterated multidimensional-liquid chromatography (TE-*Dt*-mDLC) for rapid microgram-scale structure elucidation by NMR was introduced (Ahmad et al. 2022). In a first separation dimension, mixtures are effectively separated and the peaks detected by UV are automatically trapped. Buffer is removed by a sequence of washing with H_2_O and D_2_O with independent pumps. On a second dimension the analytes are eluted with deuterated solvents and the separation is monitored by UV and MS detectors. In addition, the fractions are collected for direct NMR analysis, while bypassing drying process and minimizing analyte degradation. With this approach as little as a few micrograms of material can be analyzed and the method can achieve recoveries above 90%. It was successfully used for the characterization of indole alkaloids and synthetic compounds (Ahmad et al. 2022).

NMR is essential for the unambiguous identification of new NPs. Therefore, the different approaches to obtain this type of data quickly and efficiently from complex extracts are of great interest. High-resolution analytical and preparative techniques can be used to obtain enough pure NP for the acquisition of 1D and 2D spectra and biological assays. The choice of the most appropriate strategy depends on the sensitivity of the available NMR platforms and/or bioassays.

A direct link between the chromatographic separation and the ^1^H-NMR spectra of sequential fractions over a given chromatographic zone, or even over an entire chromatogram, allows data deconvolution and dereplication of partially co-eluting analytes. Furthermore, NMR detection on a fractionated extract provides an unbiased quantitative view of all main metabolites which typically corresponds to the LC peak detected by ELSD on state-of-the-art platforms.

Approaches such as pseudo-LC-NMR require the analysis of large numbers of generated factions, and data processing is still manual. The challenge is to integrate NMR and MS data for identification, if possible, automatically. To achieve this kind of automated identification process, it seems essential to integrate profiling data from semi-quantitative detectors such as ESLD or CAD to deconvolute the MS features of the major compounds detected by NMR.

### Green and sustainable preparative LC

Another important trend attracting some attention in the scientific literature is the greening of preparative LC. Indeed, the green chemistry movement has been exploring ways to reduce the risks of chemical exposure to humans and the environment for about 20 years. It has been estimated that the total amount of waste generated by HPLC instruments worldwide was 34 million litres per year (Gaber et al. 2011). Therefore, even an incremental improvement would result in a significant waste reduction. As mentioned in progress in sustainability in phytochemistry has been mainly in the extraction stage (Funari et al. 2023). Similar progress has not been observed in purification procedures, where chloroform, dichloromethane, and hexane are still regularly used. Several strategies have been reported for the development of environmentally friendly preparative LC techniques, some of which are discussed in this section. These include: (i) the use of alternative solvents in the mobile phase; (ii) the use of supercritical fluid chromatography (SFC); (iii) the implementation of energy-efficient processes and recycling/reuse of solvents, to minimize environmental impact.

Alternative solvents have been tested to make preparative LC greener (Claux et al. 2021). In the work of Shen et al*.*, the less toxic solvents ethanol, acetone and ethyl acetate were proposed as greener alternatives to methanol, acetonitrile and tetrahydrofuran (Shen et al. 2015). To evaluate the potential of these new solvents, five gingko terpenoids were selected as model analytes. The three alternative solvents showed significantly different selectivity, for the selected analytes. To overcome this issue and obtain a suitable separation of the model compounds, the authors developed a two-step optimization strategy combining the use of simplex design using the Snyder solvent triangle and HPLC modeling software. In the end, the minimal resolution obtained for this separation using classical and green solvents was increased from 3.13 to 5.76, highlighting the interest in using the less toxic mobile phase. When the analytical method (2.1 mm I.D.) was transferred to the semi-preparative scale (22 mm I.D. column), chromatographic purity was found to be excellent (> 99.5%), confirming that less toxic and cheaper solvents can go hand in hand with higher productivity and less waste.

Alternatively, an innovative type of solvent, known as natural deep eutectic solvents (NADES) could be considered. NADES are solvents formed by a eutectic mixture of two or more components, typically consisting of a hydrogen bond donor (e.g., an organic acid) and a hydrogen bond acceptor (e.g., an amine). When these two species are mixed, the resulting hydrogen bond network produces a substance with a lower melting point than its individual components. These solvents have been investigated as alternatives to traditional organic solvents due to their low toxicity, biodegradability, and ability to dissolve a wide range of compounds. NADES have been widely used in sample preparation, but their application in preparative LC remains very limited, mainly due to their relatively high viscosity (Dai et al. 2015; Li et al. 2015; Sutton et al. 2018; Tan et al. 2016). Besides NADES, alternative bio-based solvents, such as limonene or 2-methyltetrahydrofuran, which are readily available and renewable, can also play an important role in replacing more classical solvents, although reports for their application in chromatography in the literature are still scarce (Prache et al. 2016).

Another green strategy is to use supercritical fluids instead of liquids as mobile phase. In this case, the technique is called SFC and can be coupled with different detection modes (UV, ELSD, MS…)(Losacco et al. 2019). Due to the inherent properties of supercritical fluids, SFC can be considered as a hybrid technique between GC (high diffusivity and low viscosity) and LC (high dissolving capability), allowing high resolution, fast analysis and green separation (Desfontaine et al. 2015). In SFC, retention and selectivity are mainly determined by the chemical nature of the stationary phase, and the sorbent can be either polar, apolar or aromatic (Lesellier and West 2015). In a recent study, the applicability of SFC as an analytical strategy was evaluated using 120 highly diverse NPs (in terms of lipophilicity, hydrogen bonding ability, acid–base properties, molecular mass, and chemical structure) that were screened on a set of 15 different stationary phase chemistries (Perrenoud et al. 2016). SFC provides a suitable solution for 88% of the compounds tested, and three stationary phases (diol, non-endcapped C18, and 2-ehtylpyridine) were highlighted as particularly polyvalent, allowing suitable elution of 101 out of 120 NPs.

The mobile phase used in SFC, consists mainly of supercritical CO_2_, which has a polarity close to that of hexane. Therefore, SFC is perfectly suited for the isolation of non-polar NPs and is still primarily considered for fatty acids, terpenes, carotenoids, and essential oils, similar to what can be done in NPLC, but without the need for hexane. In addition, adding polar organic modifiers (such as methanol) to the CO_2_ mobile phase increases the elution strength, allowing the isolation of more polar metabolites (xanthones, flavonoids, alkaloids), that are commonly analysed/isolated by RPLC. For example, Vicente et al*.* have developed a method for the isolation of carnosic acid from rosemary extracts, based on semi-preparative SFC conditions. It involves the combination of a 2-Ethypyridine (2-EP) stationary phase and a mobile phase composed of CO_2_ and ethanol (Vicente et al. 2013). More recently, there has been a trend towards the addition of water to the mobile phase, to adapt SFC to the analysis of even more polar compounds, including those adapted to HILIC. For example, different types of saponins have been successfully analysed/isolated using a mobile phase containing a significant amount of methanol, and some additives (formic acid, TFA, ammonium acetate, and water) (Huang et al. 2018). SFC has thus the potential to be used for the analysis of polar and apolar extracts, thus potentially replacing several other chromatographic modes (NPLC, RPLC, and HILIC), using green and sustainable mobile phase components (van de Velde et al. 2020). SFC is also a powerful technique for preparative purposes. Indeed, it allows much better and faster separations than in NPLC, it is very convenient to use (the final evaporation of the solvent is quite simple) and it is a much greener alternative to NPLC and RPLC. In recent years, there have been at least three excellent reviews highlighting the advantages of SFC for the analysis and purification of NPs (Hartmann and Ganzera 2015), including plants used in traditional Chinese medicine (Eisath et al. 2018; Huang et al. 2018). Last but not least, SFC is also considered today as the best strategy for chiral separation at the analytical scale, and above all preparative scale, using polysaccharide-based stationary phases (De Klerck et al. 2012; Kaplitz et al. 2022; West 2019).

For greener isolation approaches it is also possible to implement energy-efficient processes to reduce energy consumption during the purification process. For example, operating parameters such as flow rate, temperature stationary phase chemistry and dimensions, or mobile phase composition can be optimized, while idle time can be minimized (stack injections) to improve the overall energy efficiency. Solvents can also be recycled/reused to minimize environmental impact and waste generation in preparative LC. However, the latter approach is not easy to implement for NPs as gradient mode is generally used.

When using an environmentally friendly solvent in the mobile phase, it is important to remember that there is always a trade-off between the performance to be achieved and the sustainability benefits of the method used. Furthermore, although green HPLC is a worthwhile approach, it may not be applicable to all types of NPs. Some applications may require the use of specific solvents or conditions that cannot be easily replaced by greener alternatives.

When considering SFC as an alternative to preparative scale LC, there are also some limitations that need to be considered. Firstly, SFC is more complex and expensive to set up than LC system. This is mainly due to the need for a specialised pump and back pressure regulator. In addition, the choice of mobile phase is quite limited compared to LC since CO_2_ must be the main component. In SFC, unlike HPLC, it is not possible to find a generic chemical stationary phase suitable for a large number of NP applications, so it is usually necessary to screen several columns. Therefore, due to the time required for these optimisation steps, the technique is mainly used for repetitive purification of well-defined samples. Optimisation of SFC methods can be more challenging than LC due to the complex interplay between pressure, temperature, mobile phase composition and stationary phase. Finally, it becomes important to recycle CO_2_ when using preparative SFC, especially at industrial scale, to have a sufficiently green and cost-effective technique, as the production of high-purity CO_2_ can also be expensive.

## Conclusions and perspectives

The recent advancements in analytical chemistry and metabolomics, specifically concerning metabolite profiling with UHPLC-HRMS, enable the analysis of natural extracts to an unparalleled degree of accuracy and precision. With the obtained on-line spectroscopic data, the relevant LC peaks can be identified and isolated with efficacy.

The advanced isolation techniques detailed in this paper illustrate that it is feasible to achieve similar high chromatographic resolutions at the preparative scale as those accomplished at the analytical level. Furthermore, the integration of MS detection and semi-quantitative data into multi-detection platforms at the preparative level permits the isolation of LC peaks of interest in a rapid and efficient manner. Computational methods can further optimise separations.

The effective integration of these new approaches into workflows for isolating NPs leads to significant rationalisation of the process. Our review aims to showcase how the latest preparative chromatography techniques can efficiently obtain pure NPs from complex biological matrices, frequently in a single step and via suitable extract enrichment.

An important aspect not addressed in this article, but that is relevant to the isolation of NPs, is the stationary phase's nature which plays an important role in chromatographic selectivity. Most NP separations currently involve the use of silica (normal phase) and C18 (reversed phase). However, natural product chemists should remain open to exploring alternative stationary phases that may better suit their specific needs. The implementation of the suitable stationary phase alongside the discussed technologies should further improve the performance of the desired NP purification.

As a perspective, such a rational approach will accelerate the discovery of specific chemical markers or new bioactive NPs. Additionally, the ability to perform structural elucidation and biological evaluation on a microgram scale allows for the study of scaffolds present at trace levels in crude extracts. These capabilities significantly broaden the boundaries of natural product research, offering new opportunities for findings in the field.
